# Extended Cleavage Specificity of Human Neutrophil Elastase, Human Proteinase 3, and Their Distant Ortholog Clawed Frog PR3—Three Elastases With Similar Primary but Different Extended Specificities and Stability

**DOI:** 10.3389/fimmu.2018.02387

**Published:** 2018-10-16

**Authors:** Zhirong Fu, Michael Thorpe, Srinivas Akula, Gurdeep Chahal, Lars T. Hellman

**Affiliations:** Department of Cell and Molecular Biology, Biomedical Center, Uppsala University, Uppsala, Sweden

**Keywords:** neutrophilic granulocyte, serine protease, hematopoiesis, proteinase 3, N-elastase, amphibian, neutropenia, phage display

## Abstract

Serine proteases are major granule constituents of several of the human hematopoietic cell lineages. Four proteolytically active such proteases have been identified in human neutrophils: cathepsin G (hCG), N-elastase (hNE), proteinase 3 (hPR-3), and neutrophil serine protease 4 (hNSP-4). Here we present the extended cleavage specificity of two of the most potent and most abundant of these enzymes, hNE and hPR-3. Their extended specificities were determined by phage display and by the analysis of a panel of chromogenic and recombinant substrates. hNE is an elastase with a relatively broad specificity showing a preference for regions containing several aliphatic amino acids. The protease shows self-cleaving activity, which results in the loss of activity during storage even at +4°C. Here we also present the extended cleavage specificity of hPR-3. Compared with hNE, it shows considerably lower proteolytic activity. However, it is very stable, shows no self-cleaving activity and is actually more active in the presence of SDS, possibly by enhancing the accessibility of the target substrate. This enables specific analysis of hPR-3 activity even in the presence of all the other neutrophil enzymes with addition of 1% SDS. Neutrophils are the most abundant white blood cell in humans and one of the key players in our innate immune defense. The neutrophil serine proteases are very important for the function of the neutrophils and therefore also interesting from an evolutionary perspective. In order to study the origin and functional conservation of these neutrophil proteases we have identified and cloned an amphibian ortholog, *Xenopus* PR-3 (xPR-3). This enzyme was found to have a specificity very similar to hPR-3 but did not show the high stability in the presence of SDS. The presence of an elastase in *Xenopus* closely related to hPR-3 indicates a relatively early appearance of these enzymes during vertebrate evolution.

## Introduction

The neutrophilic granulocyte is the most abundant leukocyte in human blood constituting 50–75% of all white blood cells, but is also one of the most short-lived cells of our immune system. They are of central importance for our defense against bacterial infections and use both granule stored antibacterial compounds and extracellular DNA containing traps to combat bacterial infections. Four types of cytoplasmic granules have been identified within neutrophils: the specific granules, the azurophilic granules, the gelatinase granules, and the secretory vesicles (1, 2). The importance of neutrophils for our bacterial defense is reflected in its granule content (3–5). Large amounts of various antibacterial compounds, including antimicrobial peptides such as defensins and cathelicidins, lysozyme, BPI, lactoferrin, and several serine proteases are stored within these granules (1, 2, 6). Four such active serine proteases have been identified in human neutrophils: cathepsin G (hCG), N-elastase (hNE), proteinase 3 (hPR-3), and neutrophil serine protease 4 (hNSP4) (7–10). A close homolog to these serine proteases, azurocidin, is also present within these granules. Azurocidin is a potent antibacterial protein but lacks proteolytic activity due to mutations in the three amino acids of the catalytic triad of the active site (11).

Based on their primary cleavage specificities these serine proteases can generally be subdivided into chymases, elastases, tryptases, asp-ases, and met-ases. Chymases are chymotrypsin-like and cleave substrates after aromatic amino acids (aa), elastases cleave after aliphatic amino acids, primarily Val, Ala, and Ile, and tryptases after basic amino acids, Arg and Lys.

Of the four active human neutrophil serine proteases hNE is probably the most potent and also one of the most abundant. hNE shows a relatively broad elastase specificity, preferring aliphatic amino acids, Val, Ala, and Ile, in the P1 position of substrates. It cleaves a number of connective tissue substrates, which is part of its role in paving the way for the neutrophil to reach sites of infection. Patients with low circulating levels of α1-anti-trypsin, which inactivates excessive amounts of hNE, suffer from severe lung emphysema due to cleavage of connective tissue components of the lung (12–14). hPR-3, the most abundant of the four human neutrophil serine proteases, also has primary elastase activity. It is currently being investigated because of its association to pathologies seen in Wegeners autoimmune granulomatosis (15, 16). The most recently identified human neutrophil protease, NSP-4, has tryptase activity and is also found in the lowest concentration within the neutrophil. No *in vivo* substrates have been identified for this protease thus far (9, 10). hCG, is also a relatively abundant enzyme, and is probably the most extensively studied protease of the four (17). A relatively detailed analysis of hCG has previously been performed using peptide libraries where hCG was compared with its mouse counterpart mCG (18). Human cathepsin G but not mCG displays a dual specificity as both a chymase and a tryptase, where the later activity favors Lys over Arg (17–19).

Although these enzymes are relatively well characterized there are a number of important unanswered questions concerning them. Their extended specificities have never been determined in detail and almost no quantitative information concerning the importance of various positions in and around the cleavage site have been presented. Such information can be used to increase the resolution during screenings of the human genome and genomes of pathogens sensitive to these enzymes in order to identify novel *in vivo* substrates. This would serve as a tool for understanding their general roles in immunity with a particular focus in bacterial defense.

The neutrophil serine proteases have been studied quite extensively for a number of years and many potential substrates have been identified. Knockout experiments show that several of these enzymes are important for bacterial and fungal defense (20–26). Flagellin of *Pseudomonas aeruginosa* and the outer membrane protein A of *E. coli* have also been identified as two potential bacterial targets (27, 28). The roles of these proteases in bacterial defense is further supported by the finding that knocking out both CG and NE impairs the elimination of *Mycobacterium bovis*, in a lung infection model in mice (25). Experimental infections using another mycobacterial species, the human pathogen *Mycobacterium tuberculosis*, also show reduced survival rates of both single (CG) and double knockout mice (NE+CG). In another study, the killing of the *Streptococcus* is also dependent on active serine proteases (23). Here the effect appears to be a combined protease response, as individual inhibition of specific proteases did not lead to a loss in killing activity (NSP-4 was not analyzed) whereas inhibition of all three reduced killing to baseline levels (23). NE and hCG may also have indirect antimicrobial effects by their recently identified effect on blood coagulation (29). Bacteria can be trapped by the coagulation in small blood vessels and are thereby inhibited from entering tissues, which results in decreased bacterial numbers (29). It is also likely that they have functions in modulating immune responses by cleavage of cytokines and chemokines.

Although the roles of neutrophil proteases, and in particular NE and CG in antibacterial defense, as well as NE and PR-3 in the cleavage of connective tissue components facilitating neutrophil access to the site of infection are relatively well-established, their extended specificities have never been determined in detail and no systematic bioinformatic screenings for potential substrates have been performed. In order to close this gap in our understanding of these enzymes we here present a detailed analysis of the extended cleavage specificities of two of them, the hNE and hPR-3. During these studies we also found that hPR3 was remarkable resistant to SDS which can facilitate the studies of its activity in cell samples without interference by activity of other proteases when analyzed in the presence of 1% SDS, as all other enzymes then are essentially inactive. To bridge the gap in our understanding of the biological role of these enzymes, we also recently published a similar study of hCG (30). In addition, and in order not to limit the study only to the specificity of the human neutrophil proteases but to obtain a more multifaceted view of these important enzymes in neutrophil biology as well as studied the presence of these enzymes in non-mammalian vertebrates and also studied a single amino acid mutant of hNE that causes neutropenia. To address the first of these issues we have expressed and analyzed the specificity of the first identifiable member of this enzyme subfamily during vertebrate evolution, an ortholog of hNE and hPR-3, in an amphibian, *Xenopus* PR-3 (31). *Xenopus* PR-3 (xPR-3) showed a similar specificity as hPR-3 but was not as SDS-resistant as its human ortholog. In contrast to hPR-3 it also showed increased potency to target cleavage sites in more tightly folded structures. However, the main features of xPR-3 were very similar to its human ortholog, indicating an early appearance of elastase-like immune enzymes during vertebrate evolution.

Concerning neutropenia the point mutation in hNE clearly indicated that this mutation resulted in a misfolded protein, which killed the early developing neutrophils by accumulation of the protein in ER thereby negatively affecting the cellular secretory machinery. This accumulation most likely slowly kills the cells during their development in the bone marrow. This latter finding shows how sensitive the developing neutrophil is to even minor disturbances when the neutrophil charges its granules with potent physiologically acting mediators of inflammation. A single amino acid mutation in one of the granule proteins causes massive death within the population of neutrophil precursors resulting in neutropenia.

## Results

### The proteases of interest

**Figure 1:**
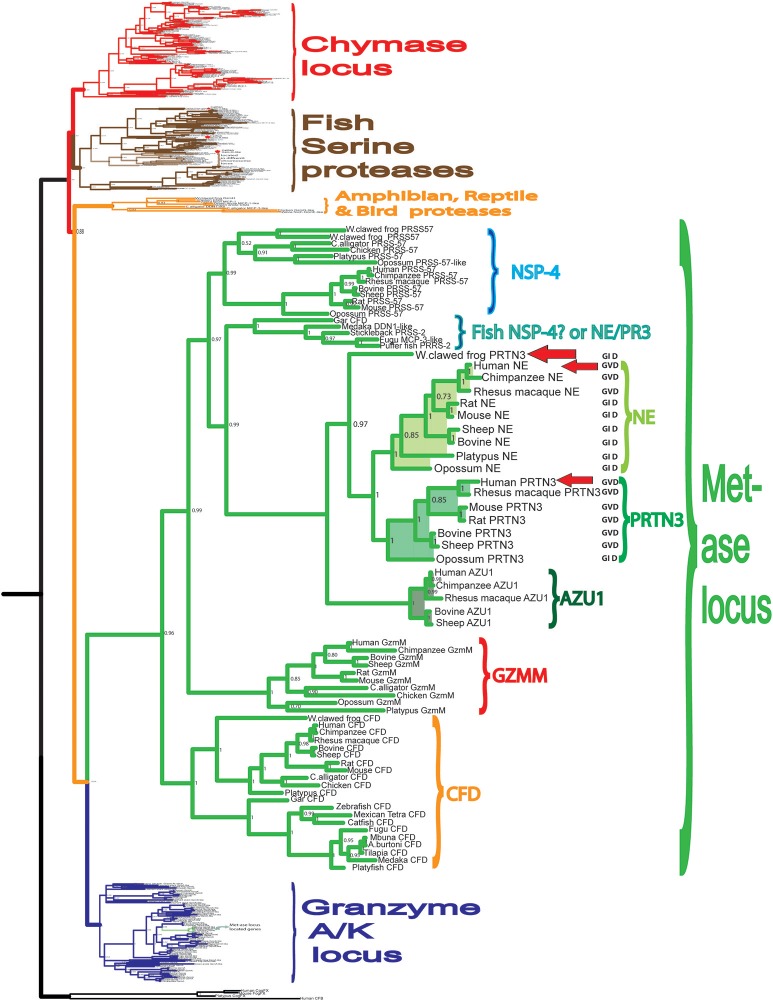
Phylogenetic analysis of the hematopoietic serine proteases. A phylogenetic tree of a large number of different vertebrate hematopoietic serine proteases using the MrBayes program. The proteases encoded within the metase locus are enlarged and highlighted and the three proteases analyzed in this communication, hNE, hPR-3, and *Xenopus* PR-3 are marked by red arrows.

To obtain a better picture of the extended specificity of the human neutrophil proteases and thereby a tool to study their biological targets we have in this study analyzed the specificity of two of the most abundant proteases of the human neutrophil NE and PR-3. Both of them are encoded from the metase locus, are structurally closely related and are thereby found in the same branch of the phylogenetic tree (Figure 1). To obtain information concerning the appearance of the neutrophil proteases during vertebrate evolution and to obtain information concerning the conservation of these enzyme specificities we have also produced a PR-3/NE homolog from the Western clawed frog, *Xenopus tropicalis* (Figure 1) (31). Amphibians represent an early tetrapod lineage with a branching point from other tetrapods estimated to be sometime around 400 million years ago. An analysis of a frog homolog could thereby give us information about whether similar enzymes existed at the time of the appearance of the tetrapods (Figure 1). From the phylogenetic tree we can see that the frog enzyme represent an early variant of these met-ase locus encoded neutrophil proteases and it is not possible from the tree to say if it is most closely related to hNE or hPR-3 (Figure 1).

The presence of a subfamily of fish proteases, which are positioned in a separate branch between NSP-4 and PR-3, NE and azurocidin is also interesting (Figure 1). This family appeared to be slightly more closely related in primary structure to PR3 and NE than to NSP-4, indicating that an early distantly related ancestor to PR-3 and NE may have been present already with the bony fishes. Furthermore, fish clearly have a homolog to mammalian complement factor D but do not appear to have a homolog to the NK cell granzyme M (Figure 1).

### The proteases

**Figure 2:**
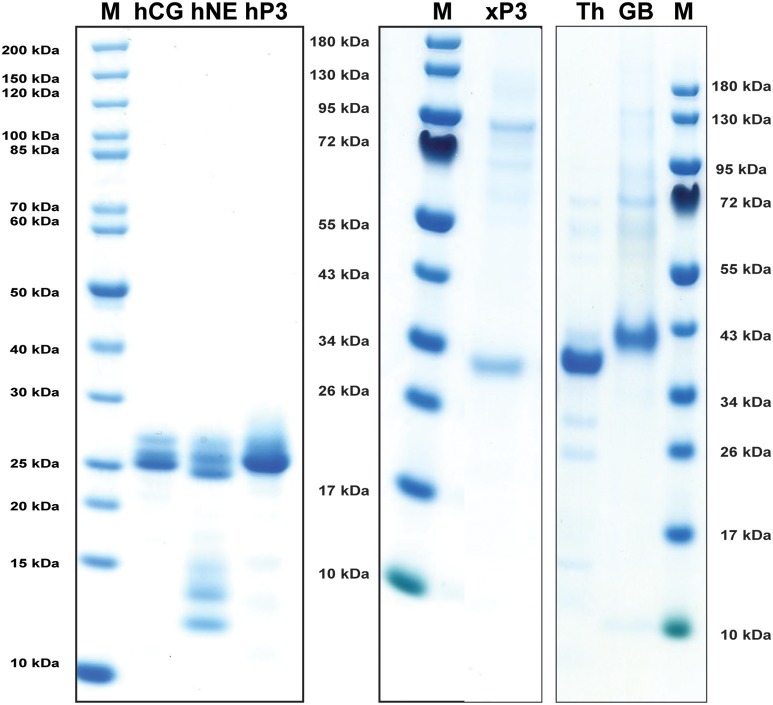
Analysis of the purified hNE, hPR-3, hCG, *Xenopus* PR-3, human thrombin (Th) and human granzyme B (GB) used in the chromogenic substrate assay and in the determination of the extended cleavage specificity. The three human neutrophil enzymes were commercial preparations purified from peripheral blood neutrophils. The *Xenopus* PR-3 was produced in the human cell line HEK293-EBNA. The xPR-3 proenzyme was first purified on Ni-NTA beads (–EK) and then activated by removal of the His_6_-tag by enterokinase digestion (+EK). The enzymes were analyzed by separation on SDS-PAGE and visualized with Coomassie Brilliant Blue staining.

Human NE, PR-3, and CG were purified from peripheral blood neutrophils. All three of them were commercially available preparations where both hCG and hPR-3 were relatively stable after purification whereas N-elastase showed unavoidable self-cleavage at a low rate even when stored at +4°C (Figure 2). *Xenopus* PR-3 was produced in the human embryonic kidney cell line HEK293 EBNA using the episomal vector pCEP-Pu2 (32). Following purification it was activated by cleavage with enterokinase to remove the His_6_-tag and the enterokinase cleavage site (DDDDK) (Figure 2). Human thrombin and human granzyme B were also used as references in the chromogenic substrate assay. Human thrombin was a commercial preparation and human granzyme B was produced in the pCEP-Pu2 vector in the human cell line HEK-293-EBNA as described for *Xenopus* PR-3.

### Chromogenic substrate assays

**Figure 3:**
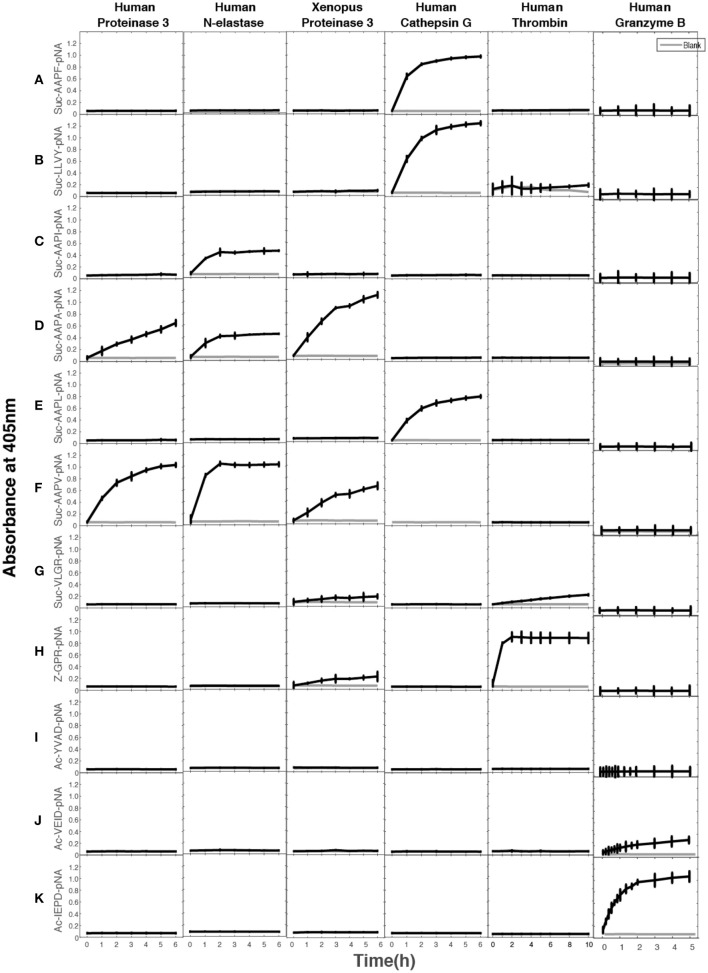
Chromogenic substrate assay. **(A–K)** A panel of different chromogenic substrates was used to determine the primary specificity of hNE, HPR-3, xPR-3, hCG, human granzyme B, and human thrombin. The panel included different chymase, elastase, tryptase and aspase substrates. The amino acid sequences of the substrates are listed on the left side of the figure. Human thrombin, hCG, and human granzyme B were included as reference enzymes for tryptase, chymase, and asp-ase activities, respectively.

A large panel of different chromogenic substrates was used to determine the primary specificities of hNE, hPR-3, and xPR-3. In order for complete specificity coverage the panel included different chymase, elastase, tryptase, and asp-ase substrates. hNE cleaved all three elastase substrates, having Val, Ala, and Ile in the P1 position (Figure 3). hPR-3 showed good activity against the Val substrate, lower activity on the Ala substrate and no activity on the others substrates, including one with an Ile in the P1 position (Figure 3). *Xenopus* PR-3 showed the best activity against the Ala substrate, lower activity on the Val, and similarly to hPR-3 no activity against the Ile substrate (Figure 3). A low tryptase activity was seen for xPR-3 (Figure 3). This activity was caused by a residual amount of active enterokinase in the preparation. As reference proteases for this assay, three additional serine proteases, hCG, human thrombin and human granzyme B, were tested. hCG cleaved the classical chymase substrates with Phe and Tyr efficiently, as well as the substrate with a Leu in the P1 position. As expected human granzyme B only showed activity toward the Asp substrate and human thrombin against the tryptase substrate with an Arg in the P1 position (Figure 3).

### Determination of the extended cleavage specificities by substrate phage display

To obtain an unbiased view of the extended cleavage specificities of the two human enzymes we performed a screening for the most favored targets using substrate phage display. The phage library used to determine the extended cleavage specificities of hNE and hPR-3 contains ~5 × 10^7^ phage clones. Each phage clone expresses a unique sequence of 9 random amino acids (nonamer) on their surface, followed by a His_6_-tag in the C-terminus of capsid protein 10. The phages are immobilized on Ni-NTA agarose beads via interactions with the His_6_-tag. The purified hNE and hPR-3 were used to screen the phage library for peptides susceptible to cleavage. After the first selection step (biopanning), the released phages, which are cleaved in their unique random region, were amplified in *E. coli* and subjected to additional biopannings. Selections of phages susceptible to cleavage by the proteases, were performed over 5 biopannings, after which hNE and hPR-3 induced the release of 71 and 106 times more phages compared to a PBS control, respectively.

**Figure 4:**
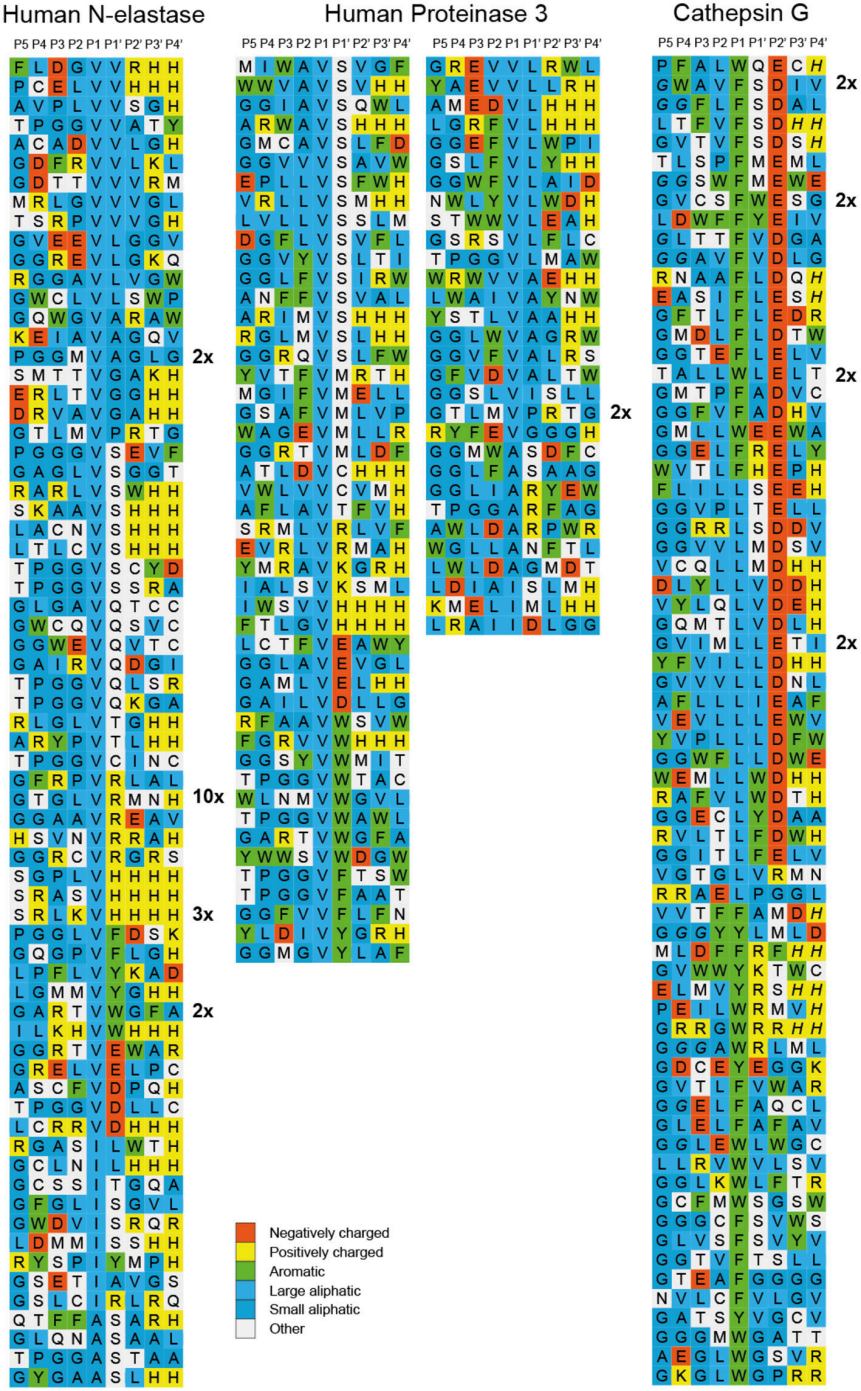
Phage displayed nonamers susceptible to cleavage by hNE, hPR-3 and hCG after five biopannings. After the last selection step, phages released by proteolytic cleavage of the three proteases were isolated and the sequences encoding the nonamers were determined. The general sequence of the T7 phage capsid proteins are PGG(X)_9_HHHHHH, where (X)_9_ indicates the randomized nonamers. The protein sequences were aligned into a P5-P4′ consensus, where cleavage occurs between positions P1 and P1′. Sequences occurring more than once are marked by the corresponding number to the right of the sequence. The aa are color coded according to the side chain properties as indicated in the legend.

After the last biopanning, 120 individual phage clones were isolated and the sequences encoding the randomly synthesized nona-peptides were determined for 96 of them (one full 96-well plate). The nucleotide sequences of good quality were then translated into nona-peptides, which were aligned based on the primary cleavage specificity observed from the chromogenic substrate assays (Figures 3, 4). Both enzymes showed a strong preference for Val and Ala in the P1 position. hNE but not hPR-3 also cleaved substrates with Ile in the P1 position quite efficiently. The alignment of both of these proteases is not easy as the sequences selected during the phage biopannings often contain numerous aliphatic amino acids, including Val, Ala, and Ile. However, the general pattern that emerges from this analysis suggests that both enzymes prefer multiple aliphatic aa (blue) both upstream and downstream of the P1 cleavage site. For hPR-3 we also saw a relatively high amount of aromatic amino acids (green) within the selected sequences, both upstream and downstream of the tentative cleavage site (Figure 4). For both enzymes, but slightly more pronounced for hPR-3, we also saw a preference for Ser in the P1′ position, where both enzymes appeared to tolerate both basic (positively charged) and negatively charged amino acids downstream of the cleavage site.

The phage display does not give the exact cleavage position, therefore the putative cleavage site was based on the chromogenic substrate assay results. The alignment of the sequences from the phage display was further refined using the information obtained from the analyses with large panels of recombinant substrates described in the next section. The alignment was thereby based on several independent assays to increase the accuracy.

### Verifying the consensus sequence by the use of a new type of recombinant protein substrate

**Figure 5:**
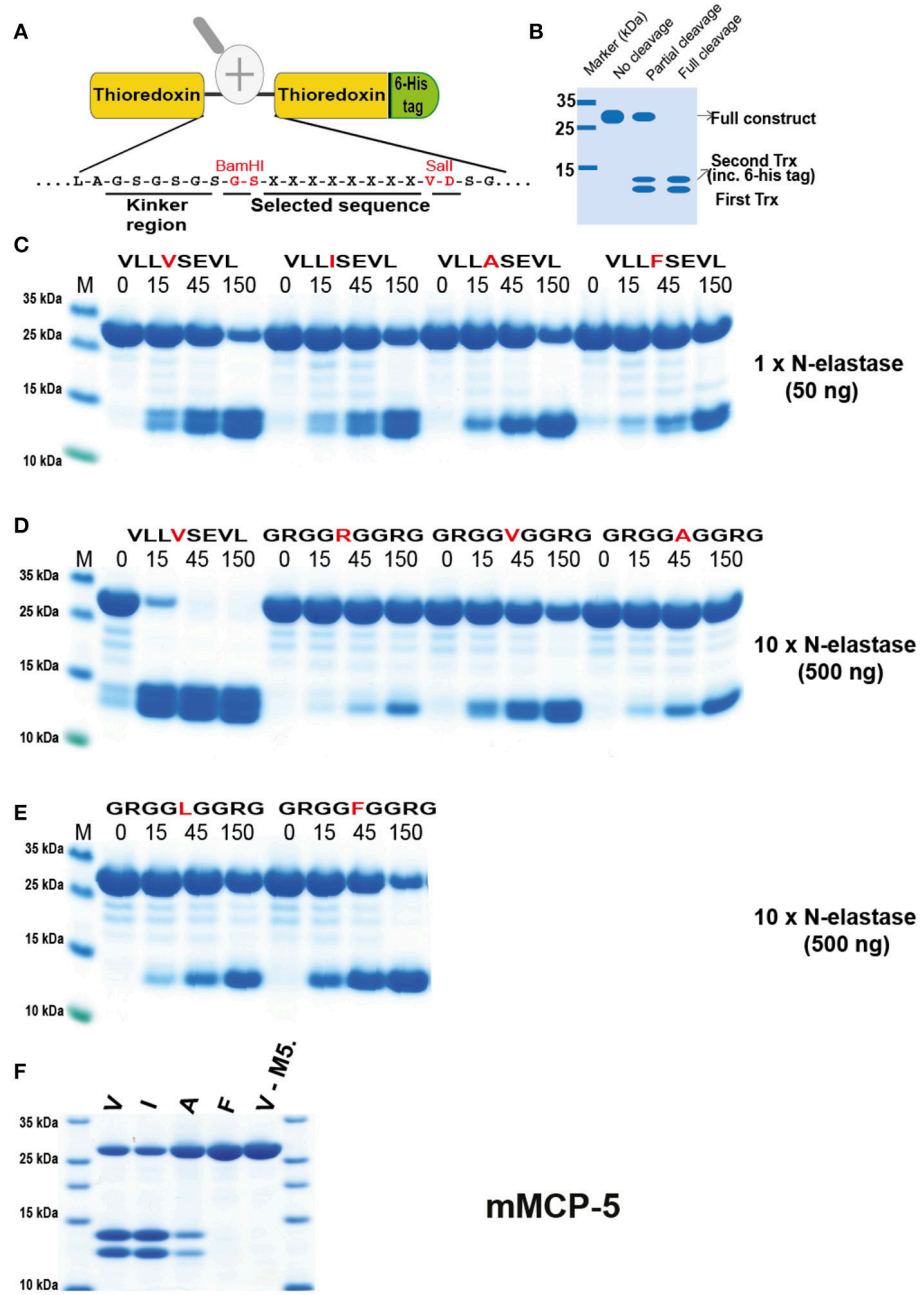
Analysis of the cleavage specificity of hNE by the use of recombinant protein substrates. **(A)** Shows the overall structure of the recombinant protein substrates used for analysis of the efficiency in cleavage by the different enzymes in Figures 5–10. In these substrates two thioredoxin (trx) molecules are positioned in tandem and the adjacent trx has a His_6_-tag positioned in its C terminus. The different cleavable sequences are inserted in the linker region between the two trx molecules by the use of two unique restriction sites, one Bam HI and one Sal I site, which are indicated in **A**. **(B)** A hypothetical cleavage is shown to highlight possible cleavage patterns. **(C–E)** Cleavage of recombinant substrates by hNE at different enzyme concentrations (50 or 500 ng). The name and sequence of the different substrates are indicated above the pictures of the gels. The time of cleavage in minutes is also indicated above the corresponding lanes of the different gels. The uncleaved substrates have a molecular weight of ~25 kDa and the cleaved substrates appear as two closely located bands with a size of 12–13 kDa. The cleavage of a panel of substrates with a more strict elastase, as represented by mouse mast cell protease 5, was included in **(F)**.

**Figure 6:**
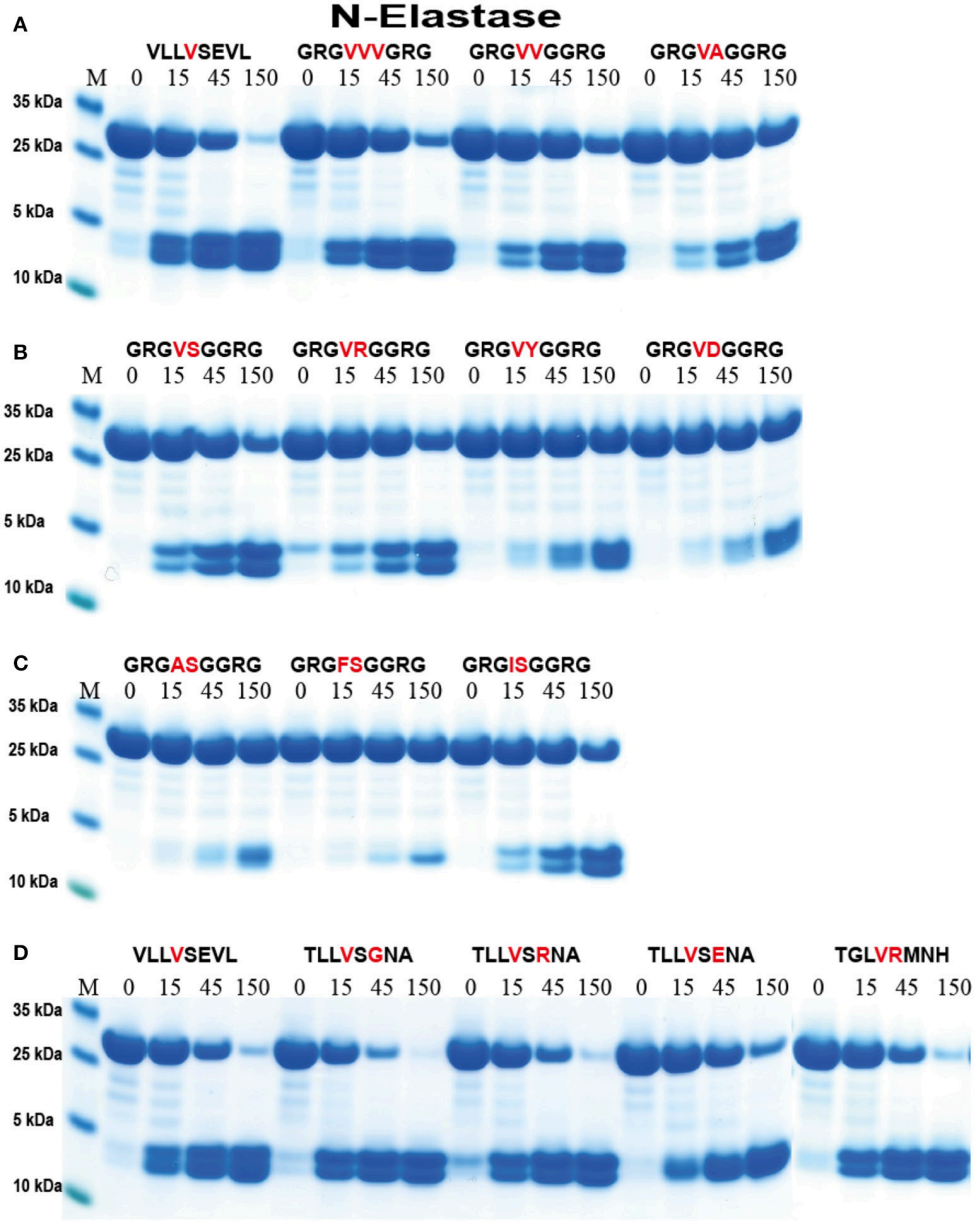
Analysis of the cleavage specificity of hNE by the use of recombinant protein substrates. **(A–D)** Shows the cleavages of a number of substrates by hNE. The sequences of the different substrates are indicated above the pictures of the gels. The time of cleavage in minutes is also indicated above the corresponding lanes of the different gels. The uncleaved substrates have a molecular weight of ~25 kDa and the cleaved substrates appear as two closely located bands with a size of 12–13 kDa.

**Figure 7:**
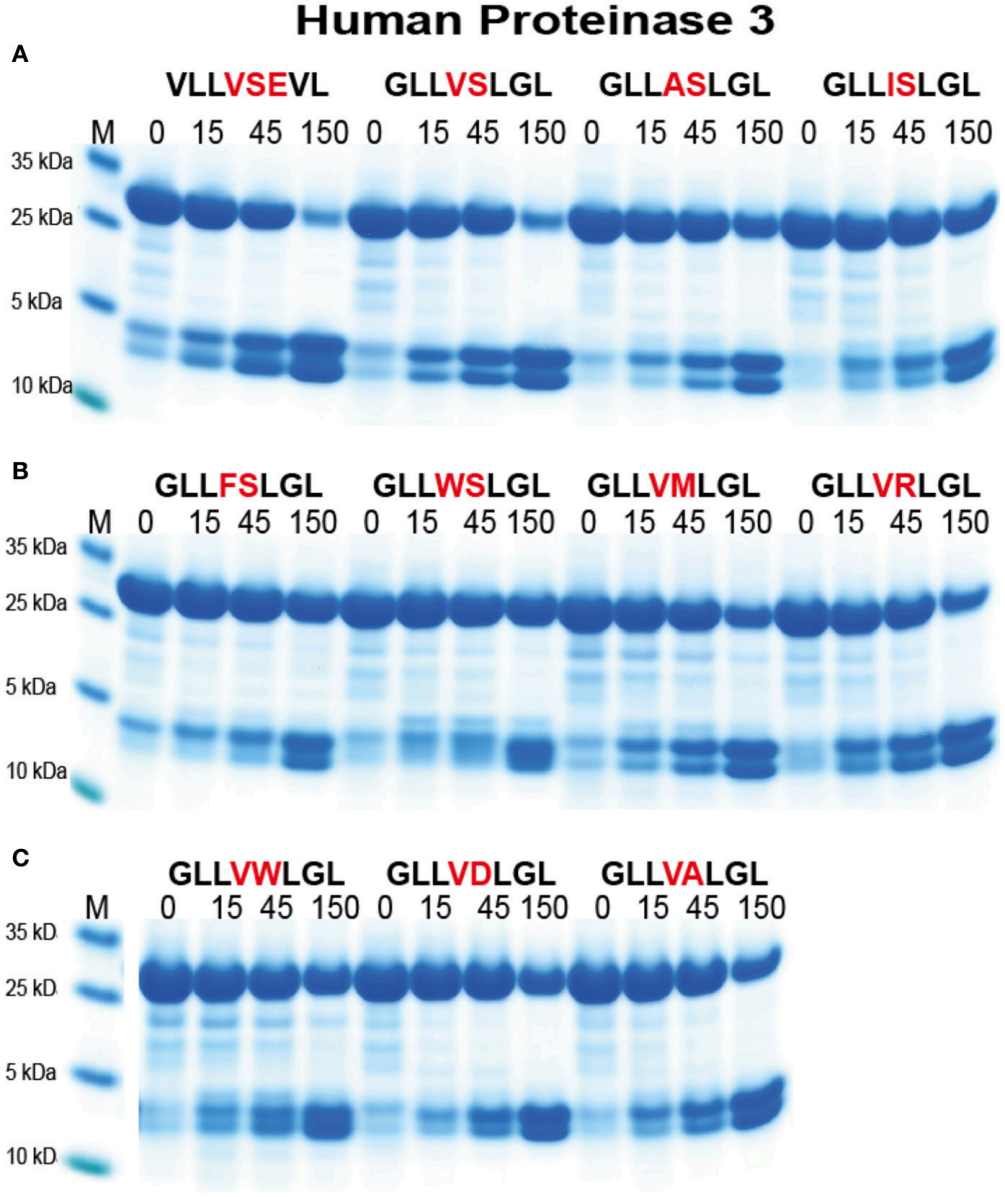
Analysis of the cleavage specificity of hPR-3 by the use of recombinant protein substrates. **(A–C)** Shows the cleavages of a number of substrates by hPR-3. The sequences of the different substrates are indicated above the pictures of the gels. The time of cleavage in minutes is also indicated above the corresponding lanes of the different gels. The uncleaved substrates have a molecular weight of ~25 kDa and the cleaved substrates appear as two closely located bands with a size of 12–13 kDa.

**Figure 8:**
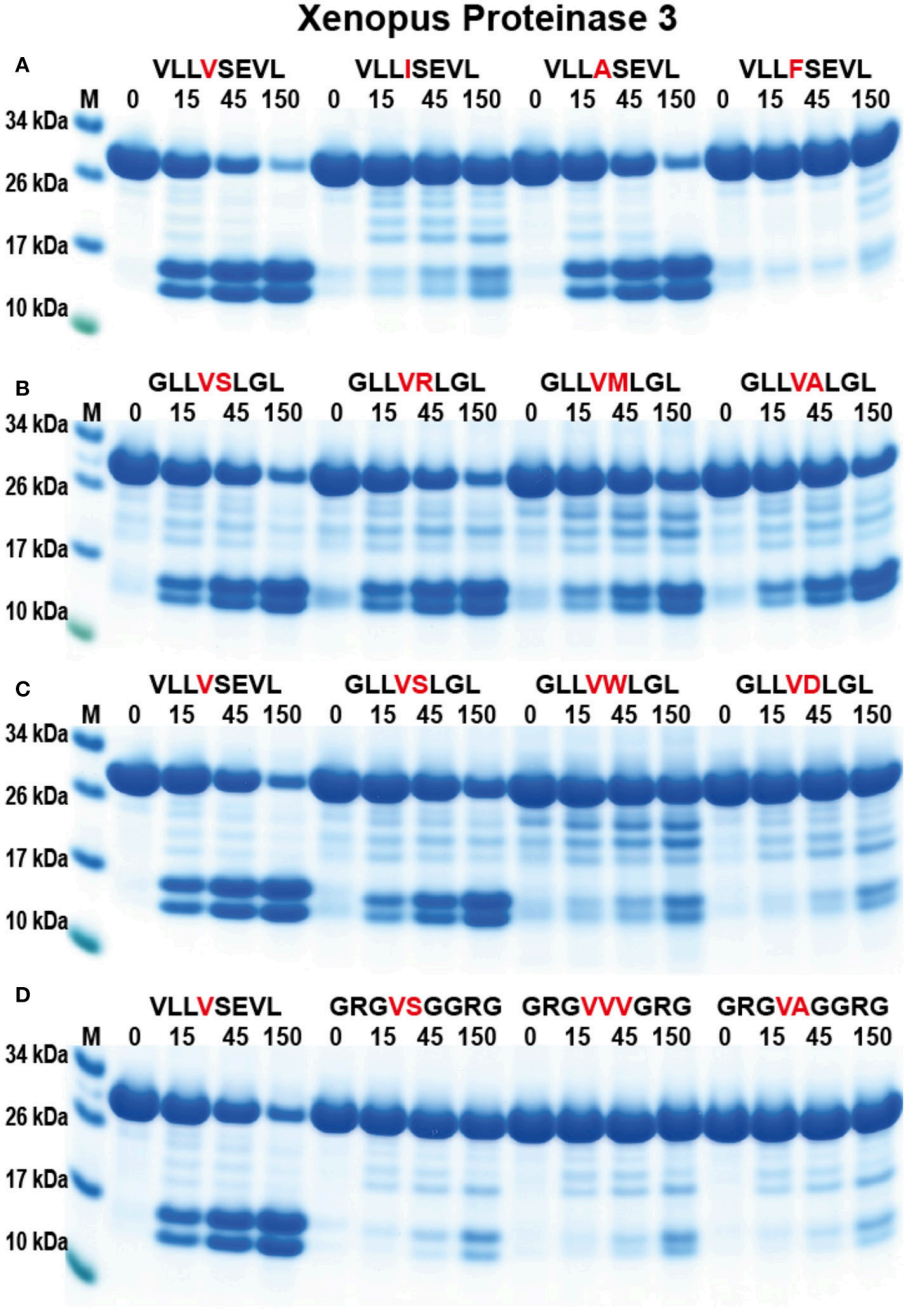
Analysis of the cleavage specificity of *Xenopus* PR-3 by the use of recombinant protein substrates. **(A–D)** Shows the cleavages of a number of substrates by hPR-3. The sequences of the different substrates are indicated above the pictures of the gels. The time of cleavage in minutes is also indicated above the corresponding lanes of the different gels. The uncleaved substrates have a molecular weight of ~25 kDa and the cleaved substrates appear as two closely located bands with a size of 12–13 kDa.

**Figure 9:**
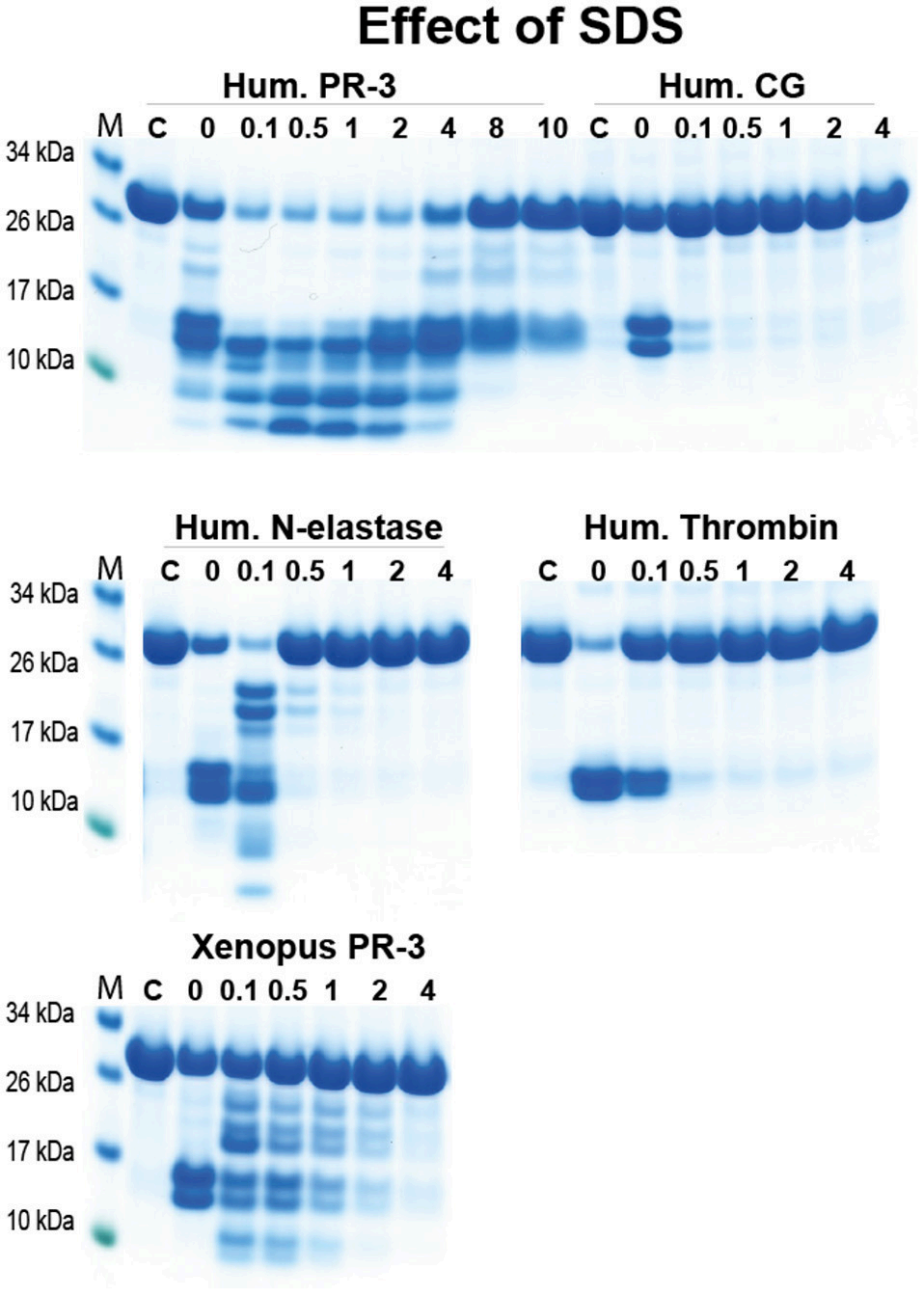
Analysis of the effect of different concentrations of SDS on the cleavage efficiency by five different enzymes, hPR-3, hNE, hCG, human thrombin and xPR-3. Consensus substrate sequences for the different enzymes were inserted in the 2x Trx substrates as shown in Figure 5. These substrates were then were used to analyze the effect of different SDS concentration on the cleavage efficiency by the five different enzymes. We used the substrate (VLLVSEVL) for hNE, the substrate (VLLFSEVL) for hCG, the substrate (VLLVSEVL) for hPR-3, the substrate (LTPRGVRL) for human thrombin and the substrate (VLLVSEVL) for Xenopus PR-3. Lane C for the different enzymes is a control without SDS. The concentrations of the SDS are then listed above the corresponding lane from 0.1 to 8%. The uncleared substrates have a molecular weight of ≈25 kDa and the cleaved substrates appear as two closely located bands with a size of 12–13 kDa. Due to the effect of the SDS to open the protein structure of the Trx molecules cleavage also occur within the Trx molecules, which results in additional bands of different molecular weights.

In order to verify the results from the phage display analysis we used a new type of recombinant substrate, which has been validated in a number of previous studies (32–38). In these substrates the consensus sequence obtained from the phage display analysis is inserted into a linker region between two *E. coli* thioredoxin (Trx) molecules by ligating a double-stranded oligonucleotide encoding the cleavable sequence into a BamHI and a SalI site of the vector construct (Figure 5A). For purification purposes a His_6_-tag was added to the C-terminal of the second Trx protein (Figure 5A). A number of related and unrelated substrate sequences were produced with this system, by ligating the corresponding oligonuclotides into the BamHI/SalI sites of the vector. All of these substrates were expressed as soluble proteins in *E. coli* and purified on IMAC columns to obtain a protein with a purity of 90–95%. These recombinant proteins were subsequently used to study the preference of hNE, hPR-3, and xPR-3 for these different sequences (Figures 5–**9**).

hNE preferred Val in the P1 position but also cleaved substrates with Ile relatively efficiently (Figure 5C). To our surprise when we introduced Ala and Phe, the enzyme seemed to prefer to cleave at the second Val further down in the sequence as seen from the difference in size of the smaller cleaved fragments. This indicated that hNE did not cleave after aromatic amino acids and does not like Ala, at least in the sequence setting of these substrates (Figure 5C). The surrounding of the P1 position also seemed to be very important. When we used substrates with repeating Arg-Gly-Gly as neighboring residues the cleavage activity was dramatically reduced (Figure 5D). Ten to fifteen times more enzyme was needed for the Val substrate (GRGGVGGRG) in order to obtain comparable cleavage seen for the (VLLVSEVL) substrate (Figure 5D). With these substrates only the Val substrate was cleaved in the center of the sequence. All the other Arg-Gly-Gly substrates were cleaved at the Val residue encoded within the Sal site of the construct (Figure 5A). Interestingly, the original P1 position thereby becomes a P6 residue and then the enzyme seemed to prefer larger aa such as Phe over both Ala and Leu in this upstream position (Figures 5D,E).

The strategy and rationale used for the selection of substrates in the analysis was based on a consensus substrate obtained from the phage display. Subsequently the surrounding residues that were found at low frequencies were used to determine how important such residues are for the cleavage of the substrate. This led to hypothetical scenarios, which we felt provided the most valuable and interesting information, for example, whether small amino acids in the neighborhood of the P1 residue reduce cleavage activity or if positive or negative charged amino acids in the near vicinity of the P1 have an effect on cleavage activities. An adjusted systematic approach was built upon the results of these types of questions, eventually covering a wide range of varied amino acids around the P1 site.

This importance of surrounding residues was assessed in greater detail (Figure 6). Having two or three Val residues in the center of the Arg-Gly-Gly substrates resulted in cleavage almost as efficient as the VLLVSEVL substrate (Figure 6A). The double VV was cleaved slightly less efficiently and the VA even less efficiently compared to the triple VVV substrate (Figure 6A). This indicates that the enzymes do not favor small aa such as Gly at positions close to the P1 site. We then continued the analysis by looking at different aa in the P1′position. There we could observe a preference for Ser and Arg over Tyr and Asp, indicating that aromatic and negatively charged amino acids are not favored in that position (Figure 6B). Interestingly neither AS nor FS was cleaved at the central position and only minor cleavage was observed at the Val of the Sal site. In contrast, the IS clone showed good cleavage in the central position, again showing that Ile in the P1 position serves as a good substrate (Figure 6C). Next we looked at the influence of the residue in the P2′ position and the result showed that the most optimal of the three substrates tested (Glu, Gly, Arg) was Gly, indicating that small aa may be favored further away from the cleavage site (Figure 6D).

For the analysis of hPR-3 we used a similar set of substrates as for hNE (Figure 7). hPR-3 appeared to also prefer Val over the other aliphatic aa, but in contrast to hNE, cleaved Ala containing substrates better than substrates with Ile in the P1 position. In contrast to hNE, hPR-3 also relatively efficiently cleaved substrates with aromatic aa in the P1 position (Figure 7B). hPR-3 seemed to be less sensitive to aromatic and negatively charged aa in the P1′position compared to hNE (Figure 7C).

To study the appearance of the neutrophil proteases during vertebrate evolution and to obtain information concerning the conservation of these enzyme specificities we have produced a PR-3/NE homolog from the Western clawed frog, *X. tropicalis* (Figure 1) (31). Using a similar set of substrates to hPR-3 and hNE, the amphibian enzyme showed a clear similarity in its specificity to hPR-3 (Figure 8). xPR-3 showed, similarly to hPR3, a strong preference for Val and Ala in the P1 position and was also not particularly accepting to Ile in the P1 position. However, and in contrast to hPR3 the *Xenopus* enzyme did not accept aromatic aa in the P1 position and was in this respect, more similar to hNE. The results from the 2x Trx substrates also indicated that xPR-3 had a slightly more restricted specificity than hPR-3.

### Analysis of the resistance of the neutrophil proteases to denaturation by strong detergents

During the sample preparation after cleavage for the SDS gel analysis of 2x Trx substrates we observed a quite strong resistance to denaturation with SDS for hPR-3. This finding indicated that we could selectively analyse the activity of hPR-3 even in the presence of numerous other proteases with similar specificities. To more closely look into this possibility, we ran cleavage reactions of hPR-3, hCG, hNE, human thrombin, and xPR-3 in the presence of different concentrations of SDS. hPR-3 was remarkably resistant to SDS and efficiently cleaved the recombinant substrates even at a SDS concentration of 4% (Figure 9). All other enzymes except xPR-3 were at a concentration of SDS of 1% completely inactivated (Figure 9). This showed that we could easily analyse the activity of hPR-3 in complex samples and even in the presence of several other enzymes without interference when the analyses were performed in the presence of 1% SDS. From this study we also concluded that xPR-3 was relatively SDS resistant, but somewhat less compared to its human counterpart (Figure 9).

### Cleavage specificity determinations by other methods—a comparative analysis

**Figure 10:**
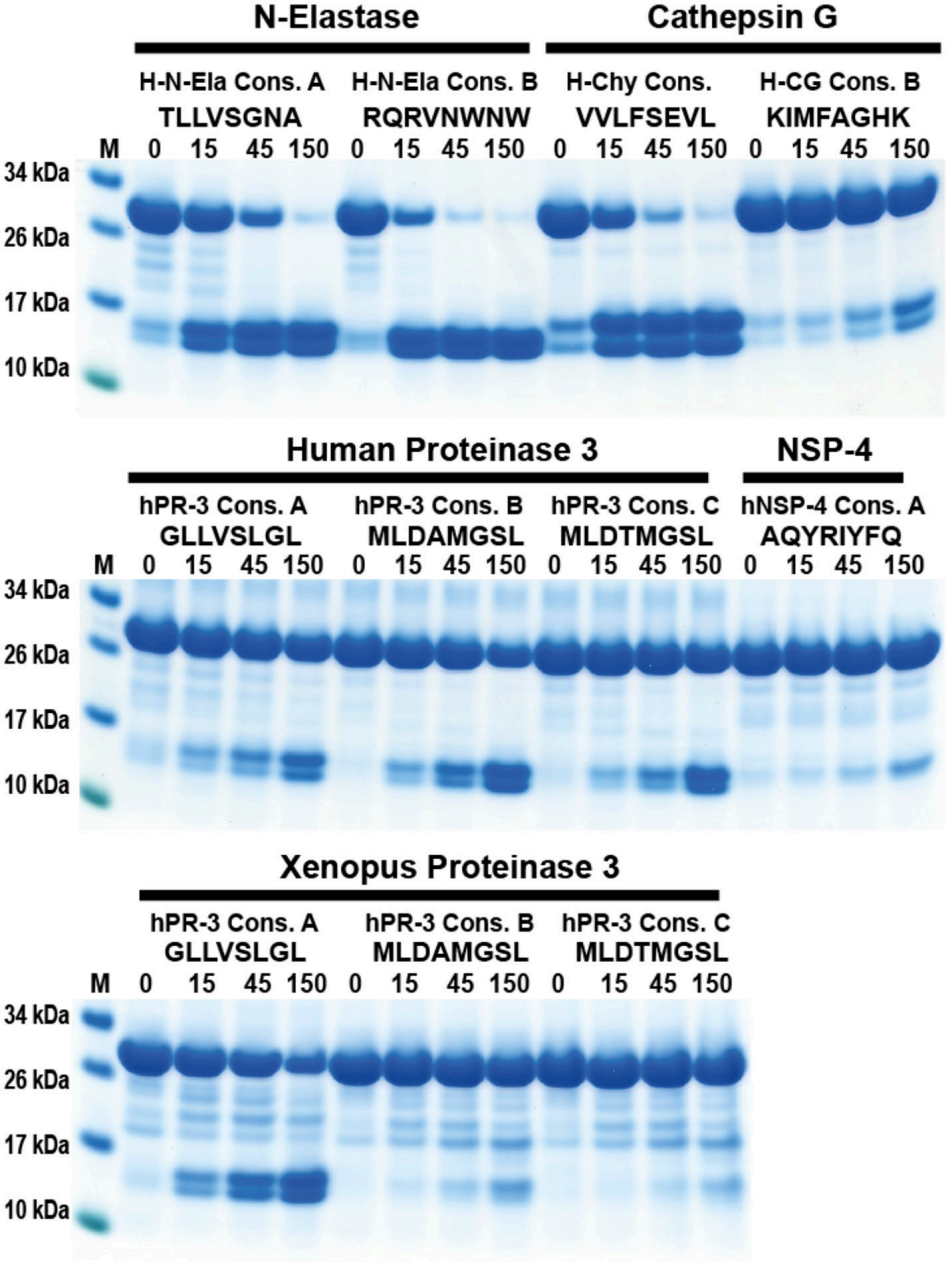
Analysis of the difference in efficacy in cleavage of a number of consensus substrates identified through phage display and an MS-based method for four human neutrophil proteases. The cleavage of a number of consensus cleavage sites for four different human neutrophil proteases (N-elastase, hCG, hPR3, and NSP4) were studied with the 2x-Trx system. The “A” consensus sites come from phage display analyses performed in our lab and the substrates B and C comes from a proteomics study by O'Donoghue et al. (39). The substrates originating from the two different methods for N-elastase and proteinase 3 both show very good cleavage, whereas for hCG the consensus sites obtained by the proteomics method shows only minor cleavage, indicating a relatively poor site. No phage display has yet been performed on hNSP4, therefore only a proteomics site was studied where minor cleavage after using a relatively high concentration of the enzyme was seen.

Recently, an alternative method to study cleavage specificity has been developed using a peptide library with 124 peptides and subsequent analysis of cleavage products by mass spectroscopy (MS) (39). In order to study the advantages and disadvantages of this method we have analyzed the cleavage of the identified consensus cleavage sites using our novel trx-based cleavage assay, with four different human neutrophil proteases: hCG, hNE, hPR-3, and hNSP4. Here, we saw that for proteases with a relatively broad specificity, such as hNE and hPR-3, the peptide library/MS method provided similar results to our analyses (Figure 10). However, for more specific proteases such as hCG, the phage display results were much more reliable as they originate from peptide libraries several orders of magnitude larger in complexity compared to the proteomics method used (Figure 10). However, one very positive characteristic of the proteomics method was the identification of substrates that were not optimal but still cleaved relatively efficiently, such as the cleavage of Thr-containing substrates by hPR3 and Lys-containing substrates by hCG, which had not been observed during the phage display screening as this method primarily identifies the most optimal substrates.

### Analysis of the effect of a single point mutation in hNE that causes neutropenia

Recently a single aa mutation in hNE (Ala to Ser in position 28 of the mature enzyme) has been identified in a German family (40). This mutation has been shown to be closely linked to neutropenia in four generations of that family (40). All members of the family that carry this mutation show different levels of reduction in their levels of blood neutrophils. In order to study the mechanism behind this effect we tried to produce both the wild-type and the mutant hNE in HEK293-EBNA cells by transfection. In several attempts to produce the protease, the wild-type gave viable transfected cells and secreted wild-type protein that could be activated by enterokinase and showed very high elastase activity. In contrast, in three independent transfections the mutant clone did not result in any surviving cells. This finding indicated that the protein, even in its proteolytically inactive form, due to the presence of a His6-tag and the entreokinase site, was highly toxic to the cells. The most likely explanation is that it affects ER or Golgi trafficking functions, possibly by aggregation and blockage. If expression levels are reasonably high such blockage would most likely slowly kill the cells. We think this shows how sensitive these cells are for even minor disturbances in structure of these highly expressed granule proteins. Even small changes in the primary sequence can result in effects on protein solubility. Due to the high expression levels of these proteins, their aggregation can subsequently result in massive death within the population of developing immature neutrophils.

## Discussion

The granule associated serine proteases constitute a large fraction of the total granule protein of neutrophils and they are important players in the function of this abundant immune cell. These proteases have also been studied quite extensively for many years, however, except for a recent study of hCG their extended cleavage specificities have never been studied in detail (7–10, 18). Their selectivity for the different potential *in vivo* substrates they may encounter is to a large extent determined by their extended specificity, which is why such information can be of major importance for the biological functions of these enzymes.

The four active proteases of the neutrophil granules show a relatively broad range of both primary and extended specificities. Human cathepsin G is a dual chymase and tryptase, with a tryptase selectivity for Lys over Arg (17–19, 30). Both hNE and hPR-3 are elastases with selectivity for aliphatic aa such as Val and Ile or Ala in the P1 position of substrates. They show a relatively low extended selectivity, as we have seen from both phage display and analyses with recombinant substrates, indicating that they can cleave a relatively wide array of substrates. Interestingly, hPR-3 can also efficiently cleave after aromatic aa and after Thr, but compared to hNE it has a relatively low activity. To obtain the same amount of cleavage we needed to use 2 μg of hPR-3 compared to only 50 ng for hNE. The fourth active protease, NSP-4, seems to only having tryptase activity. However, its extended specificity has not yet been determined and all the substrates tested so far are cleaved with very low efficiency, indicating that it has a relatively strict extended specificity (Figure 10). The broad specificity of these enzymes, possibly except NSP-4, suggests that they are involved in a number of different activities. They have been shown to cleave connective tissue components, a function most likely to facilitate the entry and migration for inflammatory cells to reach the area of infection or inflammation (7, 12–14, 41). They can also cleave a number of cytokines to regulate excessive inflammation (7, 41). In addition, they can directly act on bacteria and other infectious agents or organisms. The range of potential targets is thereby relatively broad. However, as seen for human mast cell chymase and hCG the actual cleavage of a potential target is highly dependent on the accessibility of the target site, and many proteins are remarkably resistant to cleavage of many of these enzymes even if consensus sites are present in their primary sequence (42). The broad array of cleavage activities including elastase, tryptase, and chymase can be an important factor to take into account as a single cut of a stable, properly folded protein can open the structure for subsequent attack by the other granule proteases. The neutrophil with its many active proteases, constituting several different primary specificities as well a relatively broad extended specificities, indicates that they are able to efficiently cleave many different substrates. The fact that patients with low levels of the protease inhibitor α1-anti-trypsin suffer from severe lung emphysema is one clear indication of the potency of the neutrophil proteases. Interestingly, in a mouse model, it has recently been shown that the different neutrophil proteases collectively caused more damage to the lungs than NE did alone (43). They also seem to be important components of the neutrophil extracellular traps, where the granule proteases appear to be relatively tightly attached to the DNA of the traps (39).

In the comparative analysis of two alternative methods for determining the cleavage specificity, we found that the MS-based proteomics method with only 124 peptides was good at identifying optimal cleavage sites for relatively unspecific proteases such as hNE and hPR-3 but less accurate in studying more specific proteases like hCG and hNSP-4 (Figure 10). One very positive factor with the MS-based method was the identification of secondary specificities such as Lys for hCG and Thr for hPR-3 (39). This is a very important finding that extends our knowledge of these enzymes. Looking at the phage display results in light of the Thr specificity, we can see that the sequences for hPR-3 contain many more Thr residues compared to the hNE sequences and also more aromatic aa, indicating this extended specificity. However, due to the preference for the most optimal sequences during phage display, it makes it more difficult to spot these secondary specificities. By increasing the number of peptides and only using natural aa the MS-based technology may also become a very valuable addition to the methods used to determine the specificity of more specific proteases.

Our interest in these enzymes has recently also been focused on their evolutionary and functional conservation. As one step toward this goal, here we present the activity of an enzyme closely related in activity to hPR-3, the *Xenopus* PR-3. This *Xenopus* enzyme branches just outside of the small subfamilies of mammalian PR-3 and NE in the phylogenetic tree (Figure 1), indicating that it may be a functional homolog of human NE and or PR-3 (31). By producing the recombinant enzyme we now showed that it has a specificity that in many aspects looks like hPR-3, but with some characteristics of hNE. It is also slightly more restricted in its extended specificity compared to hPR-3. The characterization of an amphibian protease closely related to a mammalian PR-3 indicates that an ancestor of these enzymes appeared very early during tetrapod evolution. The locus encoding these mammalian enzymes is also present in fish. However, no closely related members to hNE or hPR-3 have been identified in fish, which argues for the appearance of the functional homologs of the mammalian neutrophil proteases in an early tetrapod and not in bony fish (31).

**Figure 11:**
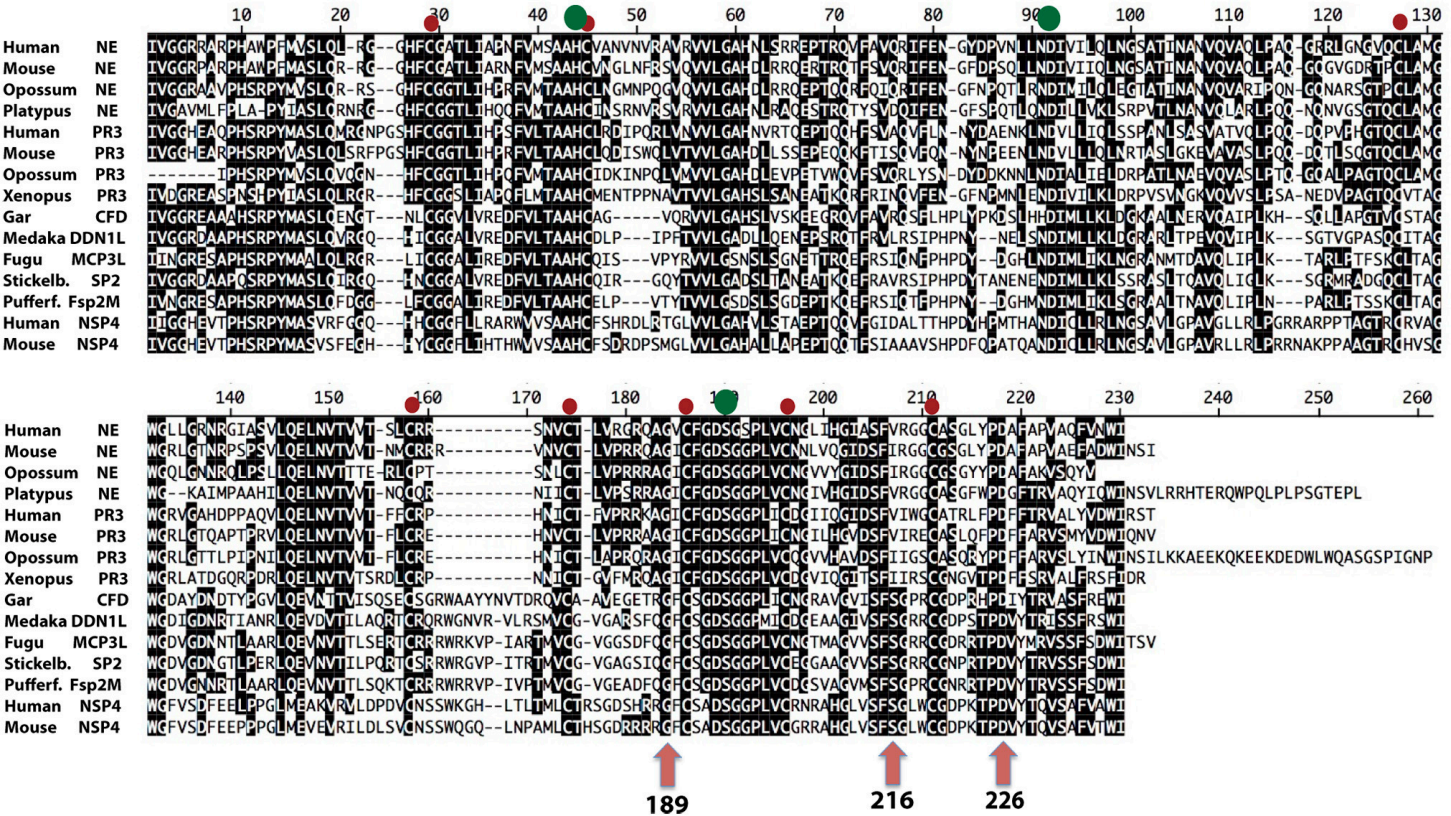
Alignment of a panel of NE, PR-3, and NSP-4 sequences. The sequences of a panel of NSP-4, NE, and PR3 sequences from mammals and amphibians were aligned using the Clustal W algorithm in the DNASTAR program together with a small subfamily of fish proteases that cluster between NSP-4 and NE and PR-3 in the phylogenetic tree shown (from Figure 1). The conserved cysteines are marked with red dots and the three residues of the catalytic triad, His-Asp-Ser, with larger green dots. The three residues with positions 189, 216, and 226 in bovine chymotrypsin are marked with purple arrows. These three residues, are in many trypsin related serine proteases, located in the bottom and the sides of the pocket of the protease where the P1 residue of the substrate is positioned upon cleavage.

In the phylogenetic tree there is a subfamily of fish proteases that map between the branch for NE and PR3, and the branch for NSP-4 (Figure 1). From the tree it actually appears as if these fish proteases are slightly more closely related in primary structure to NE and PR3 than to NSP-4, indicating that they may represent an early fish ortholog to NE and PR3 in mammals. However, from an alignment (Figure 11) we can also see that the triplet residues 189, 216, and 226, that have been shown to map to the substrate binding pocket (the S1 pocket) of mammalian enzymes, thus helping to determine the primary specificity, clearly indicate that the different fish proteases of this small subfamily are more closely related in function to NSP-4 than to NE and PR-3, as this triplet of all of them are identical to human and mouse NSP-4. Here they all have the triplet G-S-D in contrast to NE and PR-3 that have G-V/I-D (Figure 11). This may support the conclusion that the first members of elastases of the NE and PR-3 subfamily appeared with the tetrapods and that the ancestor of NSP-4 is older and may have appeared already with the bony fishes. A second possibility is that they had the same ancestor but that the mammalian enzymes duplicated and diversified into the two branches and that the branch which now harbors NE and PR-3 changed specificity and developed new functions.

One interesting aspect of hPR-3 is the very high stability in the presence of strong detergents like SDS. This was initially observed during the analyses of the recombinant substrates. When sample buffer, containing SDS, was added to stop the enzyme reaction at 15, 45, and 150 min, the amount of cleavage observed on the SDS-PAGE gels was higher at 15 and 45 min compared to 150 min. When adding beta-mercapto-ethanol, a reducing agent, the samples behaved normally, showing that breaking the cysteine bridges was needed to denature the protein. We thought this was an interesting observation as it opened the possibility of assaying hPR-3 in tissue samples without having to take into account the similar activity of the highly active hNE. By adding 1% SDS to a cleavage reaction we can now analyse the activity of hPR-3 without any interference by other neutrophil elastases. The biological relevance of this finding is not known but may indicate a higher overall stability of PR-3 compared to NE which can have an importance during infections with bacteria expressing high amounts of bacterial proteases.

We have also recently been interested in the involvement of hNE in the induction of neutropenia as a model for general neutrophil biology. One additional reason for an interest in such mutations is that a high percentage of patients with congenital neutropenia also develop leukemia. The reason for this is unknown, but is most likely partly caused by a massive proliferative response in the bone marrow of neutrophil precursors. The lack of neutrophils in the periphery make the peripheral organs send messages to the bone marrow through cytokines to produce more cells to restore normal neutrophil numbers. However, when the cells die before a mature stage the number of proliferating immature neutrophil precursors increase dramatically in the bone marrow. These proliferating cells are then susceptible targets for secondary mutations that in turn may lead to the development of leukemia. Mutations in a number of neutrophil proteins have been shown to result in neutropenia (40, 44). The three major genes are: CSF3R, which encodes the granulocyte-colony stimulating factor receptor (G-CSFR), GFI1, which encodes the growth factor independent-1 transcription repressor (GFI-1) and ELANE, which encodes neutrophil elastase (40). Mutations in the N-elastase gene are the most common, accounting for ~60% of all cases (44). Seventy-three different mutations have been identified in this gene so far. Most of these mutations are missense mutations (80%). The remaining are mutations leading to splicing defects (10%) and premature stop codons (10%). One promoter mutation has also been described (44, 45). One very interesting mutation in NE has been identified, where a single base mutation causing the change of one aa in the sequence (Ala to Ser in position 28), was found to be directly connected to neutropenia in a German family during 4 generations (40). This mutation is in a position not directly involved in the cleavage reaction as it is located far from the active site. All the persons having the mutation, homo or heterozygote, have lower levels of neutrophils and no effects are seen in other family members not carrying the mutation (40).

Our question was if this mutation still made the enzyme more active or changed the activity, thereby causing the death of the neutrophils during an early stage of maturation at the time when the gene for this protease is turned on. To address this question we transfected constructs encoding both wild-type and mutant enzymes into HEK293-EBNA cells for expression. In three independent transfections we obtained functionally active protease of the wild-type enzyme but cells did not survive with the mutant variant. No mutant enzyme could therefore be obtained for functional analyses. The enzymes are not proteolytically active when expressed in this system (due to the His_6_- and EK tags), which suggests the cause of cell death is not dependent on protease-related activity. The most likely explanation for this toxic effect is due to protein aggregation, thereby clogging the ER or Golgi. This clogging most likely severely affects the normal cell function, which finally results in cell death. In favor of this theory, the mutations in several other completely unrelated molecules have not been shown to cause neutropenia, indicating that it's not a specific protein but something that changes the behavior of the protein, possibly by exposing hydrophobic regions due to misfolding. The misfolding could then result in aggregations similar to what can be seen when heavily over-expressing proteins in bacteria where the proteins often forms inclusion bodies. A similar phenomenon is also seen during plaque formation by prion proteins. The question is now which secondary mutations within these highly proliferating neutrophil precursor cells, are the most frequent ones in the patients that develop leukemia. Such analyses would be important for potential targeting of such genes in the treatment of myeloid leukemia.

In summary, we have presented a detailed analysis of the extended cleavage specificity of two important granule proteins of human neutrophils, the serine proteases hNE and hPR-3. In addition, the specificity of one relatively closely related member of this family of proteases from an amphibian has been characterized, which showed a very similar specificity to hPR-3, indicating a relatively early appearance of these enzymes during tetrapod evolution. Interestingly, we found that hPR-3 was very resistant to detergent, making it possible to study hPR-3 activity in the presence of multiple other elastases in complex tissue environments with the addition of 1% SDS. This latter finding can come to be of importance to determine the difference in biological targets for two of the main enzymes of neutrophils, which both have elastase activity.

## Experimental procedures

### Alignment and phylogenetic analyses

#### Alignments for Figure 11

The sequences were aligned with the ClustalW algorithm in the DNASTAR program.

#### Alignments for the phylogenetic tree

The selected serine proteases were aligned in version 7 of MAFFT (http://mafft.cbrc.jp/alignment/server/), using with BLOSUM62 as the scoring matrix and a optionG-INS_I strategy for optimal results for sequences with global alignment, with default parameters (46). To check the alignment conservation and confidence the GUIDANCE2 server (http://guidance.tau.ac.il/ver2/) was used (47). To verify the multiple sequence alignment from MAFFT, another alignment algorithm (T-coffee) was used to verify that both alignments were similar.

#### Phylogenic analyses

For all proteases, the entire sequence of the active form, not including the signal sequence and activation peptide were used in the multiple alignments. The phylogenetic analyses were performed using a Bayesian approach as implemented in MrBayes version 3.1.2. Markov Chain Monte Carlo (MCMC) analyses were used to approximate the posterior probabilities of the trees. Analyses were run using the MrBayes manual standard protocol (48). The phylogenetic trees were drawn in FigTree 1.4.2 (http://tree.bio.ed.ac.uk/software/figtree/). The topologies of all Bayesian phylogenetic trees supported by posterior probabilities (PP) were verified with Bootstrap ML and distance trees using PHYLIP program package.

The PHYLIP package (v3.69) (http://evolution.genetics.washington.edu/phylip.html) was used for constructing maximum-likelihood and distance trees (49). For the distance method PROTDIST and FITCH (JTT matrix model without using an out-group species) were used. For the bootstrap analyses SEQBOOT, PROTDIST, NEIGHBOR, and CONSENSE were used to generate 100 replicate data sets from the PHYLIP package. The phylogenetic tree was drawn in Fig Tree (v1.4.2) http://tree.bio.ed.ac.uk/software/figtree/) (Figure S1). The maximum likelihood tree was generated by SEQBOOT, PROML, and CONSENSE to generate 100 replicates. The phylogenetic tree was drawn in Fig Tree (1.4.2) http://tree.bio.ed.ac.uk/software/figtree/). The different analyses looked very similar, therefore only the MrBase tree is shown in this communication. The other trees can be seen in Supplementary Figures in an earlier study of this large gene family (31).

### Production and purification of proteases

Human proteinase 3 and N-elastase were purified from peripheral blood neutrophils and purchased from Lee Biosolutions (St. Louis, Missouri, USA) and Athens Research & Technology (Athens, GA, USA), respectively. The hCG used in this study had also been purified from peripheral blood neutrophils and was purchased from BioCentrum (Krakow, Poland). Human activated plasma thrombin was purchased from Sigma-Aldrich (Sigma T-6884). *Xenopus* PR-3 and human granzyme B were produced in-house in the human cell line HEK-293 EBNA using the vector pCEP-Pu2 (32). hCG, human thrombin and granzyme B were used as reference proteases in the chromogenic substrate assays.

Protein purity and concentration was estimated by separation on 12.5% SDS-PAGE gels. Protein samples were mixed with sample buffer, and β-mercapto-ethanol was added to a final concentration of 5%. To visualize the protein bands, the gel was stained with colloidal Coomassie Brilliant Blue (50).

### Analysis of primary specificity by cleavage of chromogenic substrates

Enzymatic activity was measured toward a panel of chromogenic substrates. These substrates were purchased from Bachem (Bubendorf, Switzerland) and Chromogenix (Mölndal, Sweden). Measurements were performed in 96-well microtiter plates with a substrate concentration of 0.18 mM in 200 μl PBS. Hydrolysis was monitored spectrophotometrically at 405 nm for up to 10 h in a Versamax microplate reader (Molecular Devices, Sunnyvale, CA).

### Determination of cleavage specificity by phage-displayed nona-peptide library

A library of 5 × 10^7^ unique phage-displayed nonameric peptides was used to determine the cleavage specificity of hNE and hPR-3 as previously described (51–53). In these T7 phages, the C-terminus of the capsid protein 10 were manipulated to contain a nine aa long random peptide followed by a His_6_-tag (51). An aliquot of the amplified phages (~10^9^ pfu) were bound to 100 μl Ni-NTA beads by their His_6_-tags for 1 h at 4°C under gentle agitation. Unbound phages were removed by washing ten times in 1.5 ml 1 M NaCl, 0.1% Tween-20 in PBS, pH 7.2, with two subsequent washes with 1.5 ml PBS. The beads were finally resuspended in 1 ml PBS. PBS without protease was used as control. Phages with a random peptide that were susceptible to protease cleavage were released from the Ni-NTA matrix, and the supernatant containing these phages was recovered. To ensure that all of the released phages were recovered the beads were resuspended in 100 μl PBS (pH 7.2) and the supernatant, after mixing and centrifugation, was added to the first supernatant. To ensure that the His_6_-tags had been hydrolyzed on all phages recovered after protease digestion, 15 μl fresh Ni-NTA agarose beads were added to the combined phage supernatant and the mixture agitated for 15 min followed by centrifugation. A control elution of the phages still bound to the beads, using 100 μl 100 mM imidazole showed that at least 1 × 10^8^ phages were attached to the matrix during each selection. Ten microliters of the supernatant containing the released phages was used to determine the amount of phages detached in each round of selection. Dilutions of the supernatant were plated in 2.5 ml of 0.6% top agarose containing 300 μl of *E. coli* (BLT5615), 100 μl diluted supernatant and 100 μl 100 mM IPTG. The remaining volume of the supernatant was added to a 10 ml culture of BLT5615 (OD ~0.6). The bacteria had 30 min prior to phage addition been induced to produce the T7 phage capsid protein by the addition of 100 μl 100 mM IPTG to the culture. The bacteria lysed ~75 min after phage addition. The lysate was centrifuged to remove cell debris and 500 μl of the phage sub-library was added to 100 μl fresh Ni-NTA beads, to start the next round of selection. After binding the sub-library for 1 h at 4°C under gentle agitation, the Ni-NTA beads were washed 15 times in 1.5 ml 1 M NaCl, 0.1% Tween-20 in PBS, pH 7.2, followed by two subsequent washes with 1.5 ml PBS.

Following five rounds of selection, 120 plaques were isolated from LB plates after plating in top agarose. Each phage plaque, corresponding to a phage clone, was dissolved in phage extraction buffer (100 mM NaCl and 6 mM MgSO_4_ in 20 mM Tris-HCl pH 8.0) and vigorously shaken for 30 min in order to extract the phages from the agarose. The phage DNA was then amplified by PCR, using primers flanking the variable region of the gene encoding the modified T7 phage capsid-protein. After amplification, the PCR reactions with clearly visible fragments from 96 clones were sent unpurified to GATC Biotech Germany for sequencing.

### Generation of a consensus sequence from sequenced phage inserts

Phage insert sequences were aligned by hand assuming a preference for aliphatic aa in the P1 position. Sequences with only one or a few aliphatic aa were aligned first and sequences with more than one possible cleavage site were then aligned to fit this pattern. Amino acids with similar characteristics were grouped together as follows: aromatic (Phe, Tyr, Trp); negatively charged (Asp, Glu); positively charged (Lys, Arg); small aliphatic (Gly, Ala); larger aliphatic (Val, Leu, Ile, Pro); hydrophilic (Ser, Thr, His, Asn, Gln, Cys, Met). The nomenclature by Schechter and Berger (54) was adopted to designate the aa in the substrate cleavage region, where P1–P1′ corresponds to the scissile bond.

### Generation of recombinant substrates for the analysis of the cleavage specificity

A new type of substrate was developed to verify the results obtained from the phage display analysis. Two copies of the *E. coli* thioredoxin gene were inserted in tandem into the pET21 vector for bacterial expression (Figure 5A). In the C-terminal end a His_6_-tag was inserted for purification on Ni^2+^ IMAC columns. In the linker region, between the two thioredoxin molecules, the different substrate sequences were inserted by ligating double stranded oligonucleotides into two unique restriction sites, one BamHI and one SalI site (Figure 5A). The sequences of the individual clones were verified after cloning by sequencing of both DNA strains. The plasmids were then transformed into the *E. coli* Rosetta gami strain for protein expression (Novagen, Merck, Darmstadt, Germany). A 10 ml overnight culture of the bacteria harboring the plasmid was diluted 10 times in LB + Amp and grown at 37°C for 1–2 h until the OD (600 nm) reached 0.5. IPTG was then added to a final concentration of 1 mM. The culture was then grown at 37°C for an additional 3 h under vigorous shaking, after which the bacteria were pelleted by centrifugation at 3,500 rpm for 12 min. The pellet was washed once with 25 ml PBS + 0.05% Tween-20. The pellet was then dissolved in 2 ml PBS and sonicated 6 × 30 s to open the cells. The lysate was centrifuged at 13,000 rpm for 10 min and the supernatant was transferred to a new tube. Five hundred microliters of Ni-NTA slurry (50:50) (Qiagen, Hilden, Germany) was added and the sample was slowly rotated for 45 min at RT. The sample was then transferred to a 2 ml column and the supernatant was allowed to slowly pass through the filter leaving the Ni-NTA beads with the bound protein in the column. The column was then washed four times with 1 ml of washing buffer (PBS + 0.05% Tween-20 + 10 mM Imidazole + 1 M NaCl). Elution of the protein was performed by adding 150 μl elution buffer followed by five 300 μl fractions of elution buffer (PBS + 0.05% Tween-20 + 100 mM Imidazole). Each fraction was collected individually. Ten microliters from each of the eluted fractions was then mixed with 1 volume of 2 × sample buffer and 1 μl β-mercapto-ethanol and then heated for 3 min at 80°C. The samples were analyzed on a SDS bis tris 4–12% PAGE gel and the second and third fractions that contained the most protein were pooled. The protein concentration of the combined fractions was determined by Bio-Rad DC Protein assay (Bio-Rad Laboratories Hercules, CA USA). Approximately 60 μg of recombinant protein was added to each 120 μl cleavage reaction (in PBS). Twenty microliters from this tube was removed before adding the enzyme, the 0 min time point. The active enzyme was then added (~50 ng of the hNE and 2 μg of hPR-3) and the reaction was kept at room temperature during the entire experiment. Samples (20 μl) were removed at the indicated time points (15, 45, and 150 min) and stopped by addition of one volume of 2x sample buffer. β-mercapto-ethanol (1 μl) was then added to each sample followed by heating for 3 min at 80°C. Samples (20 μl each) were then analyzed on 4–12% pre-cast SDS-PAGE gels (Invitrogen, Carlsbad, CA, USA). The gels were stained overnight in colloidal Coomassie staining solution and de-stained for several hours according to previously described procedures (50).

## Author contributions

ZF performed experiments, produced figures, and edited text. MT performed experiments and edited text. SA performed experiments produced figures. GC performed experiments. LH designed the study, supervising experiments, writing text and designing and producing figures.

### Conflict of interest statement

The authors declare that the research was conducted in the absence of any commercial or financial relationships that could be construed as a potential conflict of interest.

## Abbreviations

hNE: human N-elastase
hPR-3: human proteinase-3
xPR-3: *Xenopus* proteinase-3.
